# Heterogeneous viromes in the salivary glands, ovaries, and midguts suggested differential viral transmission advantages of six tick genera

**DOI:** 10.1128/msphere.00540-25

**Published:** 2025-10-30

**Authors:** Jun Ni, Abulimiti Moming, Qi Chen, Yi Huang, Jian Xiao, Yang Wu, Xiaoli Wu, Chenxuan Li, Liyan Fu, Yuan Bai, Yaohui Fang, Jun Wang, Zhaojun Fan, Bo Wang, Yujiang Zhang, Feifei Yin, Jingfeng Xiong, Shaung Tang, Xuhua Guan, Fei Deng, Shu Shen

**Affiliations:** 1 State Key Laboratory of Virology and Biosafety and National Virus Resource Center, Wuhan Institute of Virology, Chinese Academy of Sciences 74614 , Wuhan, China; 2 University of Chinese Academy of Sciences 74519 , Beijing, China; 3 Xinjiang Key Laboratory of Vector-borne Infectious Diseases, Urumqi, China; 4 Center for Disease Control and Prevention of Xinjiang Uygur Autonomous Region, Urumqi, China; 5 Acute Communicable Disease Control Section, Institute for Infectious Disease Prevention and Control, Hubei Provincial Center for Disease Control and Prevention 498598, Wuhan, Hubei, China; 6 Hainan Medical University-The University of Hong Kong Joint Laboratory of Tropical Infectious Dis-eases, Key Laboratory of Tropical Translational Medicine of Ministry of Education, Academician Workstation of Hainan Province, School of Basic Medicine and Life Sciences, Hainan Medical University 12455, Haikou, Hainan, China; 7 Brain Science and Advanced Technology Institute, Wuhan University of Science and Technology 687316, Wuhan, China; 8 Hainan General Hospital (Hainan Affiliated Hospital of Hainan Medical University), Haikou, Hainan, China; 9 Vector-Borne Disease Control Section, Institute for Infectious Disease Prevention, Hubei Provincial Center for Disease Control and Prevention 498598, Wuhan, Hubei, China; 10 Hubei Provincial Center for Disease Control and Prevention 498598, Wuhan, Hubei, China; Kansas State University, Manhattan, Kansas, USA

**Keywords:** tick tissues, virome, vertical and horizontal transmission, virus dissemination

## Abstract

The transmission of tick-borne viruses is associated with the biological functions of tick tissues. This study investigated the viromes in salivary glands, ovaries, and midguts collected from nine tick species of six genera and identified 49 viruses from at least 14 families. A variety of viral abundance and diversity across the three tissues was demonstrated. Ovaries of *Rhipicephalus* (*Boophilus*), *Rhipicephalus*, *Dermacentor,* and *Hyalomma* exhibited high viral abundance, suggesting their vertical transmission advantages, while high viral abundance observed in the salivary glands of *Haemaphysalis and Ixodes* indicated their advantage for horizontal transmission. Midguts of *Rhipicephalus* (*Boophilus*), *Haemaphysalis*, *Dermacentor*, and *Ixodes* exhibited high viral diversity, as did the salivary glands of *Rhipicephalus* and *Hyalomma*. Viruses found across the three tissues (tissue-common viruses) exhibited varied abundance (confirmed by qPCR), possessing a broad tissue spectrum but reflecting different tissue preferences. Viruses presenting high abundance identified in a single tissue (tissue-specific viruses) may be indicative of limited dissemination to other tissues. Furthermore, the correlation between viral abundance and tissues indicated distinct preliminary dissemination patterns of viruses across the examined tick genera. This study demonstrated the heterogeneity of viromes in tick tissues, revealed diverse viral preferences for tissues, and suggested the transmission advantages linked to specific tick genera. The findings provide novel insight into the differential competence of virus transmission in ticks, which raised the necessity of investigation and will benefit further research into the heterogeneous landscape of viromes within tick bodies and the mechanisms for maintaining and transmitting a particular virus in specific tick species.

**IMPORTANCE:**

Tick tissues (salivary glands, ovaries, and midguts) are critical for the development of ticks and virus transmission. This study analyzed viromes in these tissues across six tick genera, thereby revealing the viral heterogeneity in tick tissues and suggesting differential transmission advantages among the examined genera. The results provide a novel insight into the understanding of the competence for virus transmission by ticks, based on viral populations, and into the mechanisms underlying virus dissemination within tick bodies, as well as those of vertical or horizontal transmission of viruses linked to the biological functions of tick tissues. The findings also suggest the importance of a more in-depth investigation into viral transmission, with a particular focus on the viromes of tick eggs, saliva, and hemolymph. All these would serve as a crucial foundational framework, establishing a robust foundation for the future development of strategies aimed at the control of viral transmission by ticks.

## INTRODUCTION

Ticks must acquire a blood meal from their hosts during their developmental stages and reproductive maturity (1). Throughout the developmental stages, tick tissues fulfill pivotal, albeit varied, functions. The most significant internal tissues in ticks include the digestive tract (consisting of the pharynx, esophagus, midgut, and rectal sac), the salivary glands, the reproductive tissues, the synganglion (fused central nervous system), the Malpighian tubules, and the tracheae. Of these, the midgut is the largest tissue, responsible for storing and digesting host blood (2). The salivary glands represent the second-largest tissue, with the capacity to secrete and produce anticoagulant factors, digestive enzymes, and proteins that promote wound formation and blood vessel dilation in the host skin (2). In female ticks that have completed their reproductive cycle, the ovary contains white oocytes of varying sizes responsible for the transfer of genetic material from one generation of ticks to the next and is the second-largest tissue after the midgut (2).

Ticks act as vectors for the transmission of viral pathogens (3–5), which are linked to the biological functions of tissues during the development of ticks. The virus can be transmitted to a vertebrate host during feeding through the secretion of saliva from the salivary glands, a process known as horizontal transmission (6). In order to achieve horizontal transmission, viruses infect and replicate in the salivary glands, creating an opportunity for secretion into the host’s blood vessels. Components in the salivary glands may facilitate the transmission of viruses, such as Thogoto virus (7) and tick-borne encephalitis virus (TBEV) (8). Tick-borne viruses can be vertically transmitted from one generation (infected adult tick) to another (larvae), thus ensuring the perpetuation of specific viruses in the life cycle of ticks (6, 9, 10). In order to achieve vertical transmission, it is necessary for the virus to be maintained in the ovaries of female ticks to increase the chance of being incorporated into eggs. Furthermore, ticks have been shown to be susceptible to viral infection when feeding on infected vertebrate hosts (11, 12). The tick may contract a virus from a host by taking blood. Once the virus establishes infection in the digestive tract, it may disseminate to other tissues such as the salivary glands and ovaries. Consequently, the virus acquired from hosts is likely to be transmitted in a horizontal or vertical manner during feeding on hosts or via egg laying.

In recent years, the development of advanced sequencing technology has facilitated the identification of numerous viruses in ticks. Research has demonstrated the remarkable diversity of tick viromes and suggested varying viral abundances among tick species (13–16). However, in previous studies, the virome of ticks was investigated through pools, providing a comprehensive understanding of the viral composition of specific ticks. Nevertheless, it remains unclear whether these virus populations are heterogeneous or uniformly distributed within ticks. Given the pivotal role of tissues in virus transmission, research into the diversity of viral populations in tick tissues would enhance our understanding of viruses with the potential to be dispersed vertically or horizontally, or to be acquired.

The present study characterized the viromes of three tick tissues, namely the midguts, the salivary glands, and the ovaries, which were collected from nine common tick species across six genera. The viral abundance and diversity among tissues were analyzed. This study compared viruses dispersed in three tissues or restricted to a specific tissue and evaluated the abundance of viruses found across three tissues. Moreover, the potential of viruses to disseminate within tick bodies was evaluated on the basis of observed associations of viral abundance between tissues. The results obtained provide insights into the tissue tropism of viruses and the competence of tissues in vectoring viruses among tick species. Consequently, virus transmission patterns for each tested tick genus were postulated, which can serve as a reference for subsequent research on the transmission mechanisms of tick-borne viruses.

## RESULTS

### Tick tissues exhibited heterogeneous viral communities among *Rhipicephalus* (*Boophilus*)*, Rhipicephalus*, *Ixodes*, *Dermacentor*, *Haemaphysalis*, and *Hyalomma*

**Fig 1:**
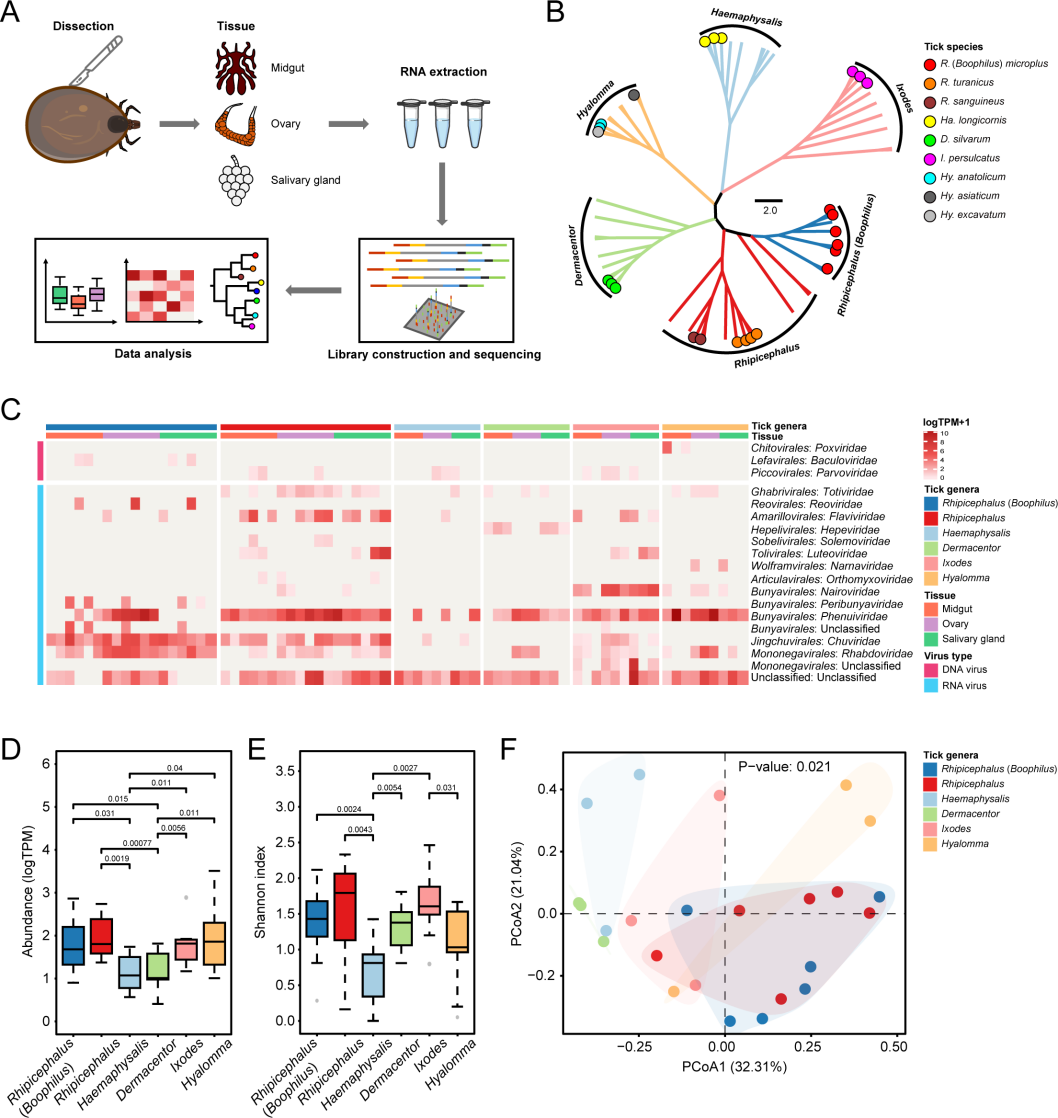
The overall viral abundance and diversity among different tick genera. (**A**) An overview of workflow in this study beginning from tissue dissection to RNA extraction, library construction, metagenomic sequencing, and data analysis. (**B**) A phylogenetic tree presenting the ticks analyzed in this study was involved in nine tick species of six genera. The *R*. (*Boophilus*) *microplus* is indicated separately from *Rhipicephalus* and is represented as an independent genus *Rhipicephalus* (*Boophilus*). (**C**) A heat map showing the abundance (viral transcripts per kilobase million [TPM]) of viral families detected in each pool. The tick genera, tissue types, and viral families are indicated. (**D**) The overall viral abundance and (**E**) Shannon index of α-diversity were compared among different tick genera. (**F**) Principal coordinate analysis (PCoA) showing the variations in viral compositions among the examined tick genera. A permutational multivariate analysis of variance (permutations = 999) was performed using the “adonis2” function. To assess the overall significant differences, the *P*-values were calculated using the Kruskal-Wallis test. In pairwise comparisons, *P*-values were calculated using the Wilcoxon rank-sum test. Outliers are shown as gray dots.

The randomly selected ticks for dissection were morphologically identified as belonging to *Rhipicephalus* (*Boophilus*), *Rhipicephalus*, *Ixodes*, *Dermacentor*, *Haemaphysalis*, and *Hyalomma*. Following dissection, the midguts, ovaries, and salivary glands were collected and prepared into 72 pools (Fig. 1A; Table S1). In contrast to other *Rhipicephalus* ticks, which are three-host ticks, *Rhipicephalus* (*Boophilus*) *microplus* is a one-host tick species. Therefore, the tissues of *R*. (*Boophilus*) *microplus* were prepared and pooled separately from the other *Rhipicephalus* ticks. The phylogenetic tree, which was constructed using the cytochrome c oxidase subunit I (*coI*) gene sequences from each pool (Fig. 1B), showed that the ticks comprised nine species across six genera: *Ixodes persulcatus*, *Haemaphysalis longicornis*, *Hyalomma anatolicum*, *Hyalomma asiaticum*, *Hyalomma excavatum*, *Dermacentor silvarum*, *Rhipicephalus sanguineus*, *Rhipicephalus turanicus*, and *R*. (*Boophilus*) *microplus*. A total of 72 pools were generated according to the tick species and locations, resulting in 24 pools for each tissue type (Table 1).

**TABLE 1:** The pools generated from tick tissues for metagenomic sequencing in this study

Tick species	Region	Number of ticks	Number of pools			
			Salivary gland	Ovary	Midgut	Total
*Dermacentor silvarum*	Inner Mongolia	99	3	3	3	9
*Ixodes persulcatus*	Inner Mongolia	193	3	3	3	9
*Rhipicephalus sanguineus*	Hainan	35	2	2	2	6
*Rhipicephalus turanicus*	Xinjiang	60	4	4	4	12
*Haemaphysalis longicornis*	Hubei	150	3	3	3	9
*Rhipicephalus* (*Boophilus*) *microplus*	Hubei	30	1	1	1	3
	Hainan	120	5	5	5	15
*Hyalomma anatolicum*	Xinjiang	29	2	2	2	6
*Hyalomma* spp.^*a*^	Xinjiang	9	1	1	1	3
Total		725	24	24	24	72

^*a*^ The pools were mixed with respective tissues from *Hyalomma asiaticum* and *Hyalomma excavatum*.

A total of 3.19 × 10^7^–1.60 × 10^8^ clean reads were generated from the 72 pools, of which 4.20 × 10^1^–1.30 × 10^6^ were related to viruses (Table S1). After *de novo* assembly, the resulting contig sequences belonged to 15 families of RNA viruses, 3 families of DNA viruses, and unclassified viruses (Fig. 1C; Table S1). The heat map revealed that the viromes of either each tick genus or the tissues of identical genus (species) exhibited distinct variations. Viruses belonging to the *Phenuiviridae* family in *Rhipicephalus*, *Ixodes*, and *Hyalomma* and those of the *Chuviridae* family in *Rhipicephalus* (*Boophillus*) were identified across the pools of all three tissues, respectively, suggesting a broad tissue spectrum of these viruses in a specific tick species. The viruses present in one or two specific tissues (e.g., *Totiviridae* in ovaries of *Hyalomma*) indicated a restricted distribution within specific tissues (Fig. 1C).

When the data from the tissues were amalgamated, the highest virus abundance was exhibited by *Hyalomma* and *Rhipicephalus*, whereas *Dermacentor* exhibited the lowest (Fig. 1D). The highest viral diversity was exhibited by *Ixodes* and *Rhipicephalus* ticks, whereas *Haemaphysalis* demonstrated the lowest (Fig. 1E). Principal coordinate analysis (PCoA) revealed that the tick pools of each genus formed discrete clusters, with the exception of *Rhipicephalus* (*Boophilus*) and *Rhipicephalus*, which formed clusters similar to each other (Fig. 1F).

### Novel viruses and new strains of known viruses are related to 14 viral families

In total, 161 viral genomes (>1,000 bp) from 49 viruses (at least 14 families) were identified from the metagenomic data. Of them, 14 novel viruses were identified and named according to the sampling locations (Table S2). Their phylogeny and sequence identities with the most closely related reference viruses, as well as the tick species from which the viruses were identified, were addressed, respectively, as follows.

**Fig 2:**
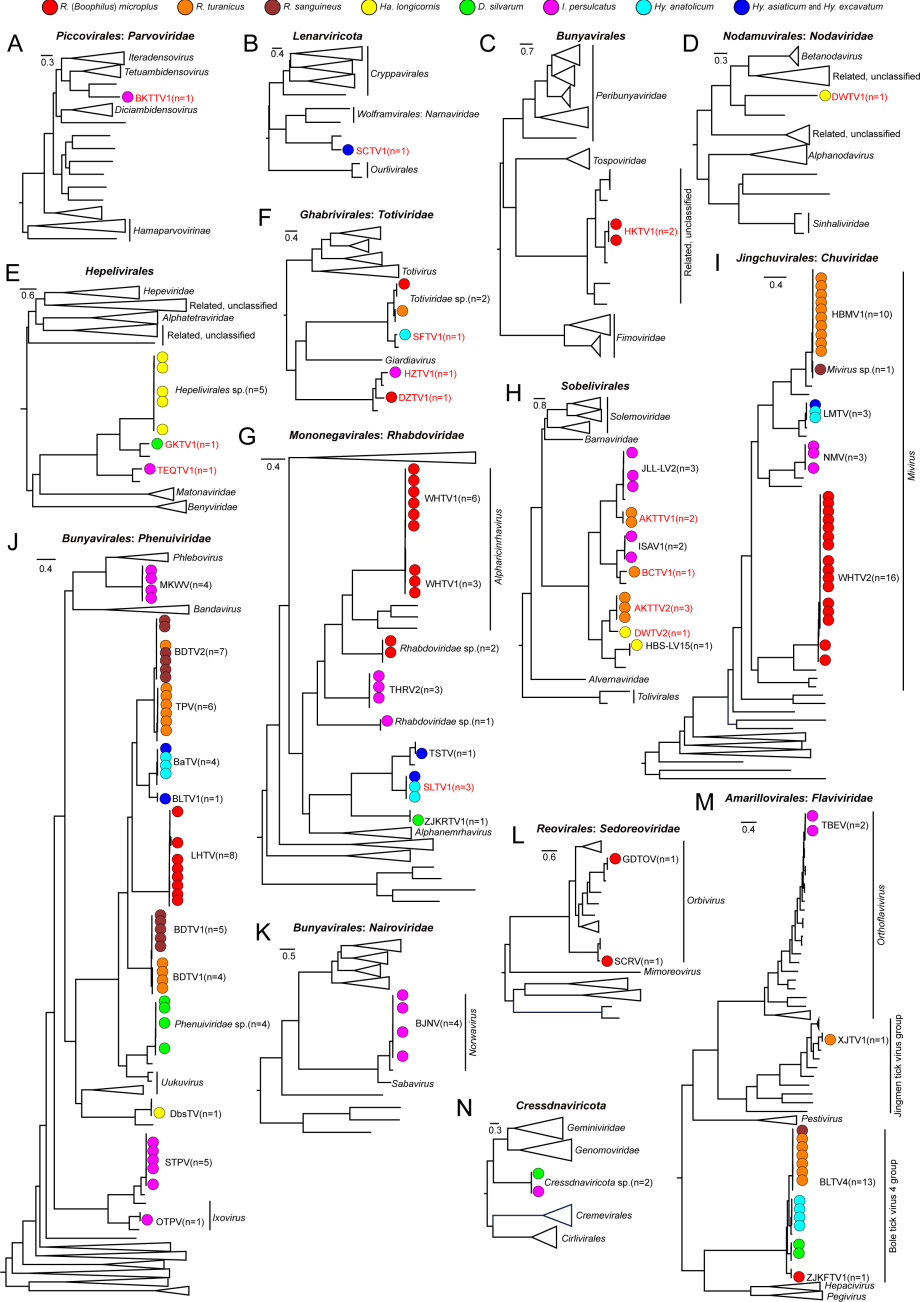
Evolutionary relationships of viruses identified in this study. Phylogenetic trees of viruses belonging to *Parvoviridae* (**A**), *Lenarviricota* (**B**), *Bunyavirales* (**C**), *Nodaviridae* (**D**), *Hepelivirales* (**E**), *Totiviridae* (**F**), *Rhabdoviridae* (**G**), *Sobelivirales* (**H**), *Chuviridae* (**I**), *Phenuiviridae* (**J**), *Nairoviridae* (**K**), *Sedoreoviridae* (**L**), *Flaviviridae* (**M**), and *Cressdnaviricota* (**N**) were constructed using maximum likelihood methods based on RdRp for RNA viruses or DNA polymerase for DNA viruses, respectively, while the tree of *Nodaviridae* was constructed using the sequences of coat proteins. The scale bars indicate amino acid substitutions per site. The viruses identified in this study are denoted with solid circles in each phylogenetic tree and colored with the tick species from which the respective virus was identified. Novel viruses are indicated in red.

Boketu tick virus 1 (*I. persulcatus*) showed 53.14% amino acid sequence identity with Lone star tick densovirus 1 (Table S2), forming a sister clade to genus *Tetuambidensovirus* in family *Parvoviridae* (Fig. 2A). Shache tick virus 1 (*Hyalomma* spp.) shared 59.27% amino acid identity with the Qingyang Narna tick virus 1 (*Narnaviridae*) (Table S2), but branched distantly related to *Narnaviridae*, suggesting a novel family of *Wolframvirales* (Fig. 2B). Haikou tick virus 1 (*R*. [*Boophilus*] *microplus*) exhibited amino acid identities of 85.88% and 75.28%–75.37% to the RNA-dependent RNA polymerase (RdRp) and glycoprotein precursor of Fuhai tick bunyavirus, respectively (Table S2), forming an unclassified clade near *Topspoviridae* and *Peribunyaviridae* of *Bunyavirales* (Fig. 2C). Dawu tick virus 1 (*Ha. longicornis*) constitutes an RNA2 segment encoding a protein with identity of 32.42% to Sanya nodavirus 1 (*Nodaviridae*) (Table S2), clustering near *Betanodavirus* (Fig. 2D). Goukou tick virus 1 (*D. silvarum*) and Taerqi tick virus 1 (*I. persulcatus*) shared 71.65% and 72.63% polyprotein identities with *Hepelivirales* sp. (*Matonaviridae*) (Table S2), forming a distinct branch that did not belong to any family of *Hepelivirales* (Fig. 2E). Three *Totiviridae*-related viruses—Danzhou tick virus 1 (*R.* [*Boophilus*] *microplus*), Hezhong tick virus 1 (*I. persulcatus*)*,* and Shufu tick virus 1 (*Hy. anatolicum*)—showed 63.3%–79.9% amino acid identities with *Totiviridae* (Table S2), but lacked genus-level classification (Fig. 2F). Shule tick virus 1 (*Hy. anatolicum* and *Hyalomma* spp.) had <72% RdRp identity to Taishun tick virus (Table S2), forming an independent branch unassigned to any genus of *Rhabdoviridae* (Fig. 2G). Four novel viruses related to *Sobelivirales* were identified: Aketao tick virus 1 (69.02% identity to Norway luteo-like virus 2), Aketao tick virus 2 (52.42%–52.69% identity to Lone star tick virus-1), and Bachu tick virus 1 (8.94% identity to Xinjiang tick virus 1) were identified from *R. turanicus*, and Dawu tick virus 2 (52.7% identity to Lone star tick virus-1) was identified from *Ha. longicornis* (Table S2). These were *Sobelivirales*-related viruses that formed a distinct clade related to other families within *Sobelivirales* (Fig. 2H).

In addition to these 14 novel viruses, viral sequences belonging to new strains of known viruses were found. The RdRp sequences of these viruses had high amino acid identities with their respective related viruses (>90%) and clustered with their reference viruses (Table S2; Fig. 2I through N).

### Disparate viral abundance and diversity in tissues indicate viral transmission advantages of the tick genera

**Fig 3:**
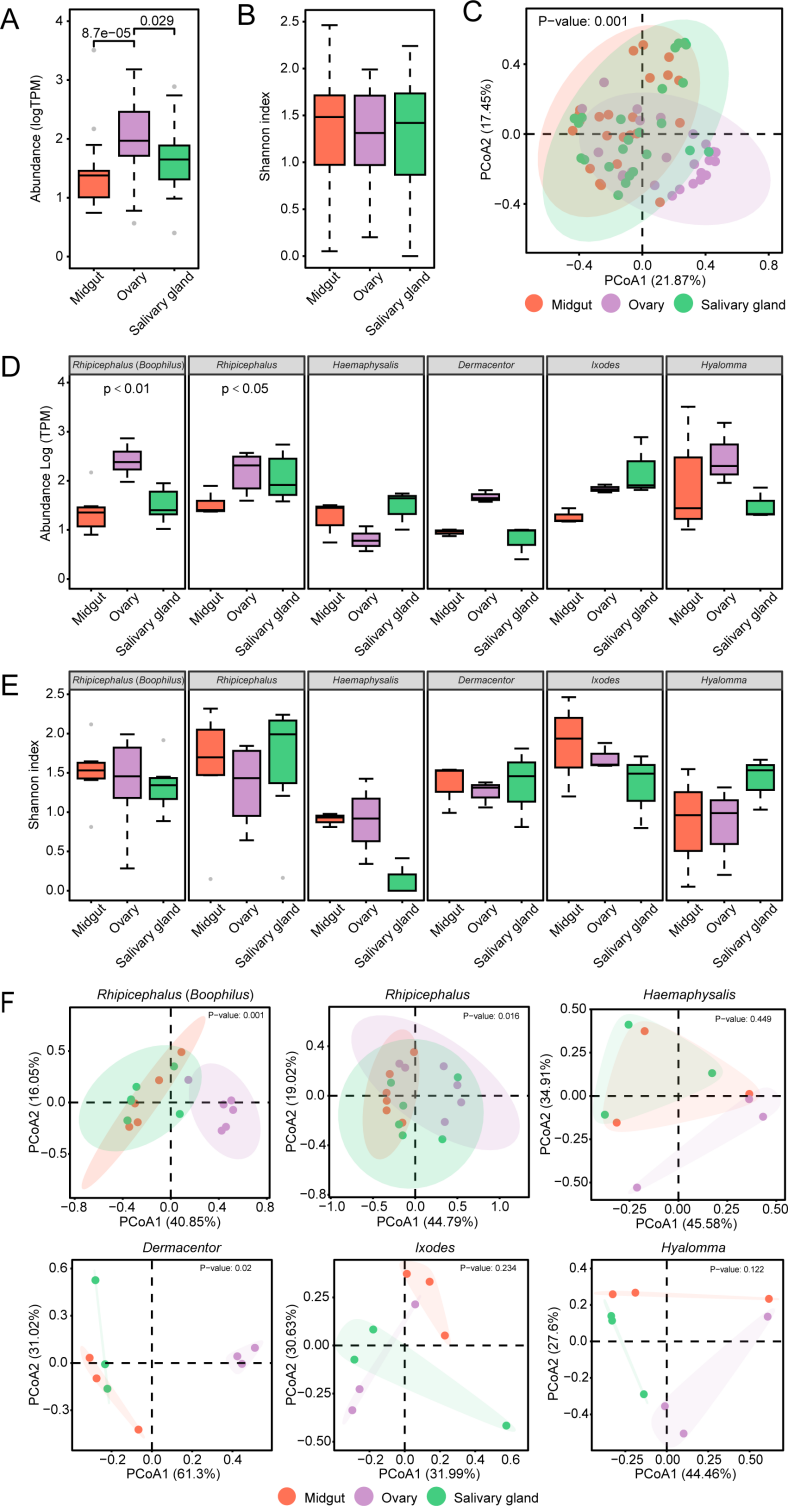
Viral abundance and diversity among tick tissues. An overall analysis of (**A**) Shannon index of α-diversity and (**B**) viral abundance of the salivary glands, ovaries, and midguts from all examined tick genera. (**C**) PCoA showing the variations of the viral compositions among the three tissues of all examined tick genera. A permutational multivariate analysis of variance (permutations = 999) was performed using the “adonis2” function. (**D**) The viral abundance and (**E**) Shannon index of α-diversity of three tick tissues in each tick genera. (**F**) PCoA presenting the variations of the viral compositions among the three tissues of respective tick genera. To assess the overall significant differences, the *P*-values were calculated using the Kruskal-Wallis test. For pairwise comparisons, the *P*-values were calculated using the Wilcoxon rank-sum test. Outliers are shown as gray dots.

Following the aggregation of data from a particular tissue type, it was evident that the ovaries manifested a significantly elevated viral abundance in comparison to the salivary glands and midguts (Fig. 3A; *P* < 0.05). The Shannon index revealed that viral diversity remained comparable across the three tissues (Fig. 3B), while the PCoA demonstrated that the viral communities in particular tissues, such as the midguts and salivary glands, were predominantly clustered (Fig. 3C). Consequently, across the tick species that were analyzed, the overall diversity of viral communities in the three tissues was comparable, while the viral abundance was generally higher in the ovaries.

The ovaries of *Rhipicephalus* (*Boophilus*) and *Rhipicephalus* exhibited the highest viral abundance, with a statistically significant difference (Fig. 3D; *P* < 0.01 and *P* < 0.05). The ovaries of *Dermacentor* and *Hyalomma* also exhibited higher viral abundances than the other two tissues, although these differences were not significant. However, *Haemaphysalis* and *Ixodes* exhibited higher viral abundances in the salivary glands than in the ovaries or midguts, although the differences were not statistically significant (Fig. 3D). The results above indicate the capability of the ovaries in *Rhipicephalus* (*Boophilus*), *Rhipicephalus, Dermacentor,* and *Hyalomma* for maintaining efficient virus infection and replication, suggesting their preference for vertical transmission. Conversely, the salivary glands of *Haemaphysalis and Ixodes* exhibited the highest viral abundance, indicating their advantage for horizontal transmission.

The three tissues exhibited no significant disparities among the tick genera that were analyzed; however, for some of the ticks, slight variations were observed. In the midguts of *Rhipicephalus* (*Boophilus*), *Haemaphysalis, Dermacentor*, and *Ixodes*, the Shannon index mean values were at their highest, indicating a high level of viral diversity. Conversely, the salivary glands of *Rhipicephalus* and *Hyalomma* exhibited higher diversity than the other tissues (Fig. 3E). PCoA revealed that the viral community structures significantly differed among the three tissues in *Rhipicephalus* (*Boophilus*), *Rhipicephalus*, and *Dermacentor* (Fig. 3F). Thus, the three tissues in the examined tick genera exhibited disparate viral compositions and diversities, albeit not to a statistically significant degree.

### The tick tissue-common viruses exhibited different tissue preferences evidenced by the viral species and abundance

We subsequently characterized the virus species found in the three tissues or identified in one specific tissue of each tick genus. Among the pools of the six tick genera, *Dermacentor* was found with the fewest viruses (*n* = 11), and *Ixodes* exhibited the most (*n* = 54). Viruses detected in a single tissue were designated as tick tissue-specific viruses, with the most viruses prevalent in *Ixodes* (30/54, 55.56%) and the fewest in *Hyalomma* (2/12, 16.67%) (Table 2; Table S3). Viruses, such as Manly virus, in the ovaries of *Rhipicephalus* (*Boophilus*), Norway luteo-like virus 2 in the salivary glands and Xinjiang tick virus 1 in the ovaries of *Rhipicephalus*, American dog tick rhabdovirus-2 in the ovaries of *Dermacentor, Ixodes scapularis* associated virus 3 in the salivary glands of *Ixodes*, and Orf virus in the midguts of *Hyalomma* (Table S3), exhibited a high abundance (transcripts per kilobase million [TPM] value > 10.00) in a single specific tissue greatly more than those of other viruses (TPM value < 10.00), suggesting that they were restricted within a specific tissue.

**TABLE 2:** The numbers and percentage of virus species found within a specific tissue and across three tissues

Tick genera	Total virus species	Tick tissue-specific viruses	Tick tissue-common viruses
*Rhipicephalus* (*Boophilus*)	20	8 (40.00%)	7 (35.00%)
*Rhipicephalus*	39	16 (40.03%)	15 (38.46%)
*Haemaphysalis*	16	9 (56.25%)	4 (25.00%)
*Dermacentor*	11	5 (45.45%)	4 (36.36%)
*Ixodes*	54	30 (55.56%)	14 (25.93%)
*Hyalomma*	12	2 (16.67%)	6 (50.00%)

**Fig 4:**
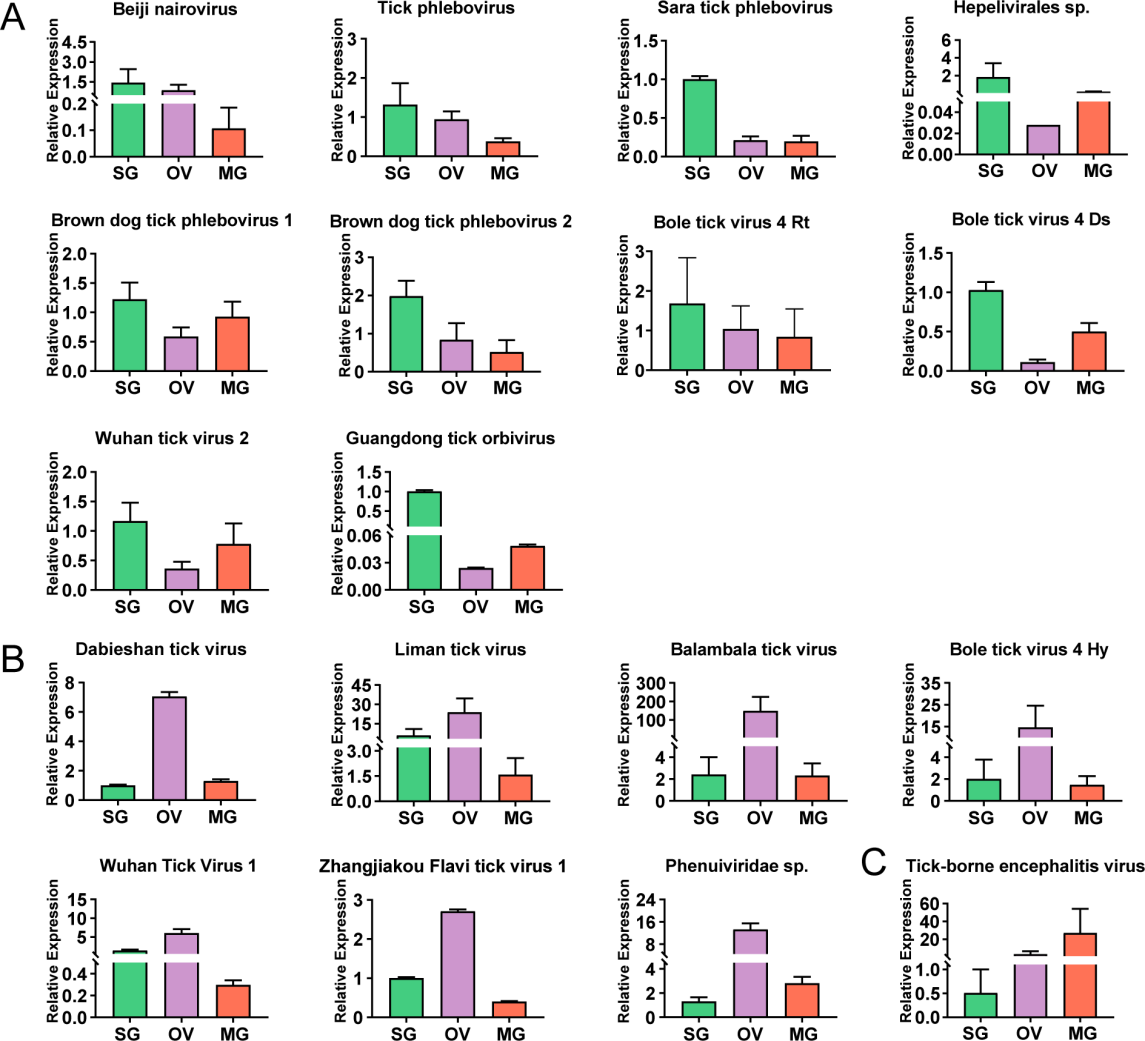
Viral abundance in salivary glands, ovaries, and midguts examined by qPCR. Based on the results of qPCR, the viruses tested were divided into three groups, which presented the highest relative expression of viral RNA in salivary glands (**A**), in ovaries (**B**), or in midguts (**C**). The relative expression levels of viral genes were normalized to that of the gene *gapdh*. The data represent mean ± SEM of two to six biological replicates according to the results of metagenomic sequencing. bole tick virus 4 Rt indicates that this virus was detected in *R. turanicus*; bole tick virus 4 Ds in *D. silvarum*; bole tick virus 4 Hy in *Hy. anatolicum*. SG, salivary gland; OV, ovary; MG, midguts.

The viruses found distributing across the three tissues were considered tick tissue-common viruses, with the most found in *Rhipicephalus* (15/39, 38.46%) and the fewest in *Haemaphysalis* (4/16, 25%) and *Dermacentor* (4/11, 36.36%) (Table 2). It is evident that some of the tick tissue-common viruses exhibited varying abundance across the three tissues, as indicated by the TPM values (Table S4) and the results of qPCR. Viruses, including Beiji nairovirus, tick phlebovirus, Sara tick phlebovirus, brown dog tick phlebovirus 1, brown dog tick phlebovirus 2, and bole tick virus 4 (BLTV4; present in *D. silvarum* and *R. turanicus*, respectively), Wuhan tick virus 2, Guangdong tick orbivirus, and *Hepelivirales* sp., were present in a high RNA load within the salivary glands more than in the ovaries and midguts (Fig. 4A). This finding suggests that these viruses were better adapted to the salivary glands of the respective tick genera. Viruses including Liman tick virus, Balambala tick virus, BLTV4 (in *Hy. anatolicum*), Wuhan tick virus 1, Zhangjiakou Flavi tick virus 1, *Phenuiviridae* sp., and Dabieshan tick virus exhibited higher viral loads in the ovaries than in the salivary glands or midguts (Fig. 4B), suggesting a preference for the ovaries of these viruses. Notably, TBEV, a pathogen that causes severe disease in humans (17), was identified in *I. persulcatus* and exhibited the highest load in the midguts (Fig. 4C), suggesting a preference for infection in this tissue or acquisition from the blood of hosts. The findings indicated that these viruses under investigation have disseminated across the salivary glands, ovaries, and midguts; however, they exhibited distinct tissue preferences.

### Virus dissemination between the primary and secondary target tissues in six tick genera

**Fig 5:**
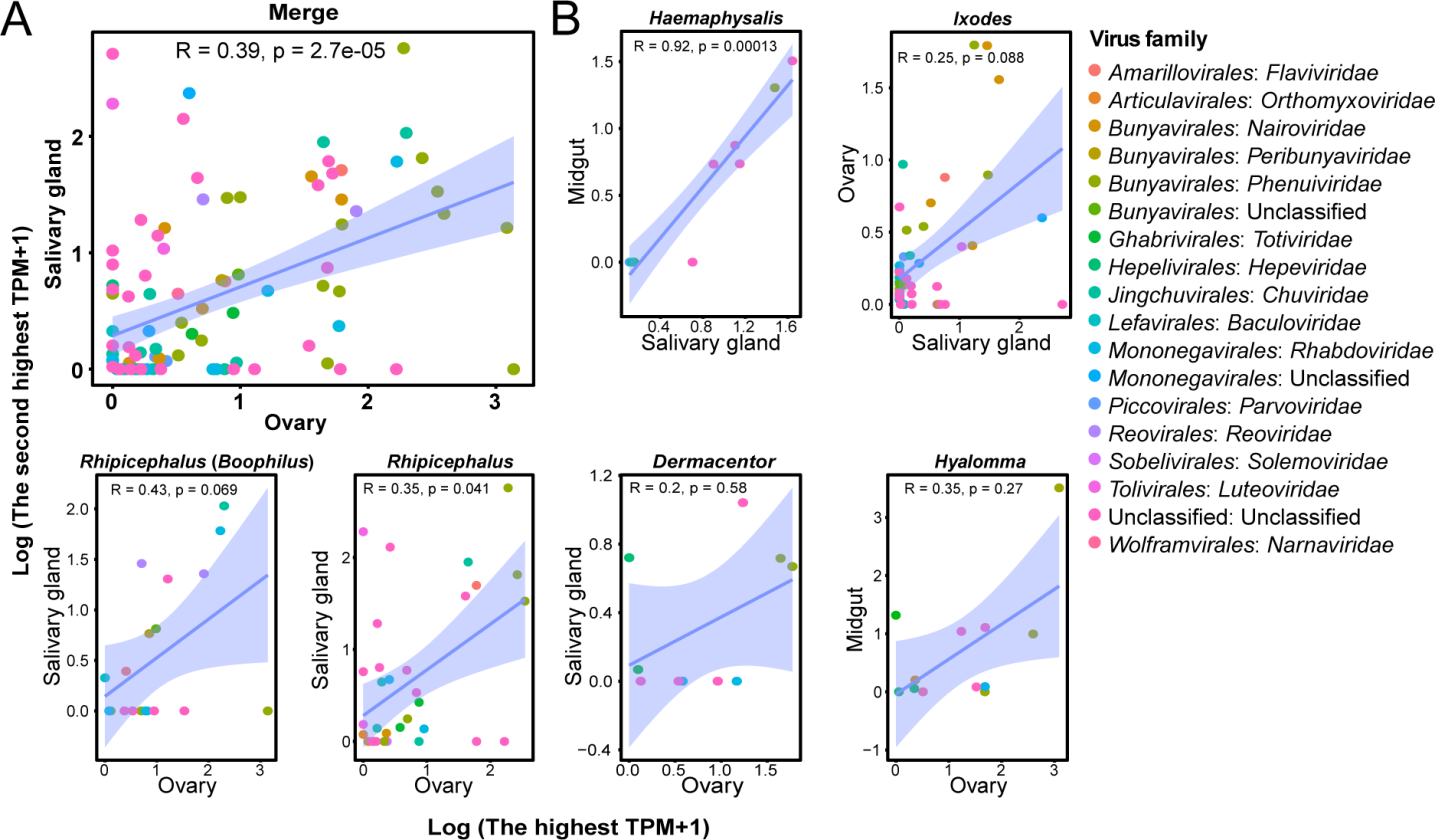
The association analyses of the abundance of viral families found both in the primary and secondary target tissues. (**A**) An overall analysis of the association between the primary and secondary target tissues of all examined tick genera. (**B**) Analyses of the association between the primary and secondary target tissues of respective tick genera. For all analyses, the primary target tissue is present along the *x*-axis, and the secondary target tissue is present along the *y*-axis. Each point represents one virus in the family.

It has been hypothesized that once the concentration of viruses within a primary tissue reaches a certain threshold, these viruses can disseminate to other tissues in small mammals and be detected (18). In this study, the primary and secondary target tissues of the examined tick genera were designated as outlined in Materials and Methods. A comprehensive analysis of the data pertaining to the tissues of all the tick genera examined showed that the ovaries were the primary target tissue, while the salivary glands were the second. A significant association of the abundance of viruses found in both tissues was observed between these two tissues, and the viruses were related to more than 17 families (Fig. 5; *P* < 0.01), indicating a general and predominant dissemination pattern from ovaries to salivary glands (Fig. 5A). This finding was similar to the genera *Rhipicephalus* (*Boophilus*), *Rhipicephalus*, and *Dermacentor* as associations were identified between ovaries and salivary glands, which incorporated viruses of 8, 10, and 4 families, respectively, with a significance level of *P* < 0.05 in *Rhipicephalus. Haemaphysalis*, *Ixodes*, and *Hyalomma* may pose divergent patterns. In *Haemaphysalis*, the primary and secondary target tissues were salivary glands and midguts, respectively, with significant association by including viruses of five families (Fig. 5B; *P* < 0.01). The primary and secondary target tissues were the salivary glands and ovaries in *Ixodes*, respectively, by including viruses from nine families. The ovaries and midguts were the primary and secondary target tissues, respectively, in *Hyalomma* by including viruses of six families, although not significantly (Fig. 5B). Taken together, *Phenuiviridae*, *Chuviridae*, and *Rhabdoviridae* constituted the core viral families found in these tick genera, yet these were related to different dissemination patterns.

## DISCUSSION

In the present study, the viromes of the salivary glands, midguts, and ovaries collected from nine tick species of six genera were characterized, which identified 161 viral genomes (>1,000 bp) from 49 viruses belonging to at least 14 families. In addition, this study focused on the diverse abundance and diversity of viral populations present in tick tissues, with the objective of enhancing comprehension of the competence of the three tissues in the context of virus transmission. In general, viral abundance in the ovaries was significantly higher than that observed in the salivary glands or midguts. The ovaries play a pivotal role in the transmission of genetic material from one generation of ticks to the next via the process of oviposition (19, 20). Consequently, the presence of viruses within the ovaries that are capable of efficient replication may facilitate their successful incorporation into eggs and ensure the perpetuation of the virus across successive generations. A high abundance of viruses was observed in the ovaries of *Rhipicephalus* (*Boophilus*), *Rhipicephalus, Dermacentor,* and *Hyalomma,* suggesting that these tick species may possess the advantage of transmitting viruses across generations through vertical transmission. During the process of blood-feeding, the salivary glands of the tick dynamically synthesize and secrete a multifaceted array of active molecules to overcome the host’s defense and to disrupt hemostasis, thereby ensuring the successful engorgement (21). Consequently, viruses that are present in high abundance within the salivary glands may facilitate their presence in saliva. Furthermore, the transmission of these viruses could be enhanced by the immunotolerant microenvironment at the feeding site that is created by saliva (22, 23). A high viral abundance was observed in the salivary glands of *Haemaphysalis* and *Ixodes*, suggesting a potential advantage of horizontal virus transmission.

Viral populations were divergent among the three tissues, as substantiated by the Shannon index analysis and PCoA, in addition to the heat map illustrating viromes. A high α-diversity of viruses was observed in the midguts of *Rhipicephalus* (*Boophilus*), *Haemaphysalis, Dermacentor*, and *Ixodes*, in contrast to the comparatively low viral abundance in this tissue. It is hypothesized that the abundance of viruses would be reduced due to the potential impediment of viral replication within the complex and hostile intestinal microenvironment, which exerts antimicrobial effects through factors such as blood flow, temperature, microbiological communities, defensin-related molecules, and hemocidins (24–28). Cross-species signaling pathways also exist within ticks. When ticks ingest blood, they simultaneously intake mammalian interferon-gamma (IFN-γ). The tick utilizes the IFN-γ receptor Dome1 to activate the Janus kinase-signal transducer and activator of transcription (JAK-STAT) signaling pathway, thereby inducing effective bactericidal activity (29). It is evident that not all viruses possess the capability to establish a robust infection and replicate efficiently in the midguts. This would lead to a state of high viral diversity but a low viral abundance. The viral diversity among the three tissues was also supported by enumerating the distinct viral species in these tissues. The tissue-specific viruses of high abundance indicated their specific preference in a particular tissue, which may be related to the route of transmission for these viruses. Consequently, the viruses only present in ovaries, including Manly virus, Xinjiang tick virus 1, and American dog tick rhabdovirus-2, may possess preference for vertical transmission, and the viruses only present in salivary glands, such as Norway luteo-like virus 2 and *Ixodes scapularis* associated virus 3 in *Rhipicephalus* and *Ixodes*, respectively, may possess a preference for horizontal transmission. Orf virus, which was found only in the midguts of *Hyalomma*, is a pathogen that causes cutaneous pustular lesions in sheep and goats and is also related to zoonotic infection in humans (30, 31). To date, no evidence exists to supports Orf virus transmission by ticks, despite the detection of this virus in *Haemaphysalis qinghaiensis* collected from skins of yaks and Tibetan sheep (32). The results obtained in this study indicate that Orf virus in the midguts of *Hyalomma* was likely acquired from animal hosts through blood-feeding. It was unable to be transmitted by ticks as being restricted within the tick intestinal tract. Furthermore, while the tissue-common viruses presented a broad tissue spectrum, they exhibited different abundance across the three tissues. These suggest that the tissue-common viruses still possessed a tissue preference, while having disseminated within the tick bodies. TBEV is a viral pathogen that is believed to be transmitted primarily by *Ixodes* ticks (33) and could be maintained in ticks via transovarial transmission (34). In this study, TBEV was identified presenting a high load in midguts more than in ovaries and salivary glands of *Ixodes*. Due to the ticks collected being blood-fed, this may have had a positive effect on the TBEV amplification in the intestinal tract. Or else, TBEV may be acquired from animal host via blood-feeding, survived, and established a robust infection in midguts, and then disseminated to the salivary glands and ovaries. Furthermore, BLTV4 was identified as present across the three tissues of *D. silvarum, R. turanicus*, and *Hy. anatolicum*. However, BLTV4 exhibited a high abundance in the ovaries of *Hy. anatolicum* (Fig. 4B), but in the salivary glands of *D. silvarum* and *R. turanicus* (Fig. 4A). This indicates a transmission preference of BLTV4 in *Hy. anatolicum* (vertically) differing from that observed in *D. silvarum* and *R. turanicus* (horizontally). Therefore, the role of tick species in the tissue preference of viruses merits further investigation. The primary and secondary target tissues were designated on the basis of the viral abundance in the examined tick genera, as previously established (18), in an attempt to characterize the potential overflow of viruses between the major tissues. Our results indicated a predominant dissemination pattern from ovaries to salivary glands in *Rhipicephalus* (*Boophilus*), *Rhipicephalus*, and *Dermacentor*. But the observed pattern was from salivary glands to midguts in *Haemaphysalis*, from salivary glands to ovaries in *Ixodes*, and from ovaries to midguts in *Hyalomma*. These findings indicate that the tick genera may possess divergent preferences in terms of virus dissemination among tissues in the examined tick genera. Nevertheless, viruses belonging to *Phenuiviridae*, *Chuviridae*, and *Rhabdoviridae* identified across the three tissues were involved in the abundance association between the primary and secondary target tissues of all examined tick genera. This suggests that viruses of these families may exhibit a robust infection and possess the capability for a broad tissue tropism in a diverse range of tick genera.

The study has limitations. First, given that the ticks were collected from the body surfaces of animals, factors such as the ecological environment, the host species, the duration of their attachment to the hosts, and the volume of blood that they have fed may have influenced the identification of viral compositions in the tissues, consequently affecting their abundance and diversity. This study did not set out to determine how environmental, host, or feeding status affects tick viromes; rather, it focused on analyzing viral abundance and diversity, and their potential correlation with the biological roles of the respective tick tissues. Second, the predominant dissemination patterns among the tissues of the examined tick genera were determined based on the sequencing data, which suggested the preference of the group of viral populations in ticks. Both virus and tick species could influence the patterns, as discussed above. Hence, it is important to investigate the transmission and dissemination properties using an experimental tick model infected with a specific virus in order to further address the mechanisms underlying viral transmission. This study also raised unsolved issues. The dissemination of viruses among tissues may rely on the hemolymph circulating within the tick body. However, due to technical limitations and operational constraints, hemolymph samples were not collected from the identical tick groups in this study. Further research into the viromes of the hemolymph of ticks from wild habitats or from experimental models is recommended. Our findings indicated the different advantages of the examined tick genera in the horizontal and vertical transmission. Investigating the viromes of tick saliva and eggs would provide further insight into the different viral populations involved in horizontal and vertical transmission, respectively.

Overall, this study demonstrated the heterogeneity of viral compositions among salivary glands, ovaries, and midguts of the six tick genera examined, as evident from the viral abundance and diversity. The results suggest that different tick genera may have advantages for virus transmission vertically or horizontally as well as virus dissemination patterns between tissues within tick bodies. These findings provide novel insights into the differential competence of the examined tick genera for virus transmission. Further investigation is strongly recommended to clarify the landscape of virus heterogeneity within tick bodies and the mechanisms of maintaining and transmitting viruses in terms of a specific virus in a particular tick species.

## MATERIALS AND METHODS

### Tick sample collection and dissection

From 2021 to 2023, ticks that parasitized animals (sheep and cattle) were collected separately during the peak seasons of tick activity in Xinjiang, Inner Mongolia, Hubei, and Hainan, China. Partially engorged live female ticks were randomly selected for disinfection. In brief, tick species were distinguished according to their morphological features by a professional technician. Ticks considered the same species or genus from the same sampling location were assigned to one group. Each selected tick was immersed in 75% ethanol thrice for 30 s each and washed thrice with sterile phosphate-buffered saline (PBS; pH 7.2). Ticks assigned to one group were dissected under a microscope at the same time to collect their salivary glands, ovaries, and midguts. Then, the tissues from one tick group were pooled separately, resulting in one pool per tissue type and three pools for a tick group. To avoid cross-contamination, the dissection tools were separately used for each tissue, and the tissue was washed thrice in fresh sterile PBS before pooling. All selected ticks were dissected immediately after being transported to the laboratory.

### Metagenomic sequencing and data analyses

Total RNA from each pool was extracted using the RNAiso Plus Reagent (TaKaRa, Shiga, Japan) as previously described (35) and was quantified using a Qubit 4.0 fluorometer (Thermo Fisher Scientific, Waltham, MA, USA). In a total volume of 40 µL of RNA from each pool, 25 µL RNA was used for metagenomic sequencing, and the remaining 15 µL was used for subsequent qPCR. After assessing the quality using an Agilent Bioanalyzer 2200 (Agilent, Santa Clara, CA, USA), RNA was fragmented and reverse transcribed into a strand-specific cDNA library using the VAHTS Universal V6 RNA-Seq Library Prep Kit (Vazyme, Nanjing, China). Paired-end (2 × 150 bp) sequencing of each RNA library was performed using an Illumina NovaSeq 6000 System.

The adapters were removed from the raw sequencing reads, which were then quality-trimmed using Trimmomatic (v.0.39) (36). Clean reads were *de novo* assembled using MEGAHIT (v.1.2.9) (37). All contigs were subjected to BLASTN (v.2.12.0+) against nucleotide databases (nt, 2023/5/12 version) using the local BLAST tool (38) and BLASTX (v.2.0.13) against nonredundant protein databases (nr, 2023/5/12 version) from GenBank using DIAMOND (v.0.9.24) (39). The *E*-value was set at 1 × 10^−5^. After excluding phages and reverse-transcribed viruses, a second round of contig assembly for those homologous to viruses was performed using SeqMan in the Lasergene package (v.7.1) (40). Reassembled contigs (>1,000 bp) contained complete or nearly complete viral genomes. The NCBI ORFfinder was used to search each of the viral genome open reading frames (https://ftp.ncbi.nlm.nih.gov/genomes/TOOLS/ORFfinder/). According to the International Committee on Taxonomy of Viruses (ICTV) criteria, an RdRp or replicase with <80% amino acid similarity to known species was defined as a novel virus and confirmed using a phylogenetic tree (18). Moreover, the tick species in each group were further confirmed by analyzing the sequences of the *co1* (~1,500 bp) gene from each pool.

### Analyses of tick virome diversity and abundance

To estimate the abundance of each virus in different pools, clean reads from each pool were mapped against the viral contigs using Bowtie2 (v.2.5.0) (41) with end-to-end alignment with default parameters. SAMtools (v.1.16.1) (42) was used to sort and index these alignments, from which the read counts for each viral contig were obtained. Viral abundance was determined by normalizing the absolute read counts using TPM, as previously described (35). The viral diversity was characterized at the viral family level. The α-diversity (within-library virome richness) and β-diversity (between-library dissimilarity) were analyzed using the vegan package (v.2.6-4) in R (v.4.3.2) based on the Shannon index and PCoA, respectively. The Kruskal-Wallis test and Wilcoxon rank-sum test were used to evaluate the statistical differences in α-diversity and viral abundance. For β-diversity, a permutational multivariate analysis of variance (permutations = 999) was performed using the “adonis2” function. Finally, the graphics were visualized using the R packages pheatmap (v.1.0.12) and ggplot2 (v.3.4.4).

### Phylogenetic analysis

Phylogenetic analyses were performed using the amino acid sequences of RdRp (RNA virus) or replicase (DNA virus) of the viruses identified in this study, together with genome sequences of the respective reference viruses downloaded from the ICTV. Phylogenetic analyses were conducted using PhyloSuite (v.1.2.3) (43). Briefly, amino acid sequences of RdRp or replicase were aligned using MAFFT (v.7.471), and the best-fit substitution model was predicted using ModelFinder. The maximum likelihood phylogenetic trees were constructed using IQ-TREE (v.1.6.8) and assessed using 1,000 bootstrap replicates. All trees were visualized using FigTree (v.1.4.4).

### Correlation analyses of viral abundance between tissues

According to the previous study (18), the tissues presenting the highest and intermediate abundance of viruses were considered to be the primary and secondary target tissues, respectively. The correlation between viral abundance in the two target tissues was then analyzed using a simple linear regression model. Spearman tests were performed using the stat_cor function of the ggpubr (v.0.6.0), and the stat_smooth function in ggplot2 (v.3.4.4) was used to fit the trend line and visualize it in R.

### Quantitative PCR

The remaining RNA (15 µL) from each tick pool was reverse-transcribed using the PrimeScript RT Reagent Kit with gDNA Eraser (TaKaRa, Japan). Real-time qPCR was performed using SYBR Premix Ex Taq II (TaKaRa, Japan) with the primers designed according to the viral sequences identified in this study (Table S5). The viral abundance in each tissue was quantified by first normalizing the Ct values to those of the glyceraldehyde-3-phosphate dehydrogenase (*gapdh*) gene in the corresponding tissue and then to the relative expression levels of respective viruses in salivary glands using the 2^–ΔΔCt^ method, as previously described (44). Graphics were visualized using the GraphPad Prism software (v.8, La Jolla, CA, USA).

## Data Availability

We submitted the meta-transcriptomic data assemblies to the NCBI Sequence Read Archive (SRA; SRR27032943, SRR30597370–SRR30597440) under Bioproject PRJNA1048150. The assembled viral genomic sequences are available in GenBank (accession numbers PV093636–PV093878).

## SUPPLEMENTAL MATERIAL

Supplemental tables msphere.00540-25-s0001.xlsx Tables S1–S5.

ASM does not own the copyrights to Supplemental Material that may be linked to, or accessed through, an article. The authors have granted ASM a non-exclusive, world-wide license to publish the Supplemental Material files. Please contact the corresponding author directly for reuse.
